# Metal-Chelating Self-Assembling Peptide Nanofiber Scaffolds for Modulation of Neuronal Cell Behavior

**DOI:** 10.3390/mi14040883

**Published:** 2023-04-19

**Authors:** Kenana Dayob, Aygul Zengin, Ruslan Garifullin, Mustafa O. Guler, Timur I. Abdullin, Abdulla Yergeshov, Diana V. Salakhieva, Hong Hanh Cong, Mohamed Zoughaib

**Affiliations:** 1 Institute of Fundamental Medicine and Biology, Kazan (Volga Region) Federal University, 18 Kremlyovskaya St., 420008 Kazan, Russia; kdaiob@stud.kpfu.ru (K.D.); tabdulli@gmail.com (T.I.A.); abdulla.ergeshov@mail.ru (A.Y.); divsalahieva@kpfu.ru (D.V.S.); 2 Scientific and Educational Center of Pharmaceutics, Kazan (Volga Region) Federal University, 18 Kremlyovskaya St., 420008 Kazan, Russia; 3 Department of Instructive Biomaterials Engineering, MERLN Institute for Technology-Inspired Regenerative Medicine, Maastricht University, P.O. Box 616, 6200 MD Maastricht, The Netherlands; a.zengin@maastrichtuniversity.nl; 4 Department of Aeronautical Engineering, University of Turkish Aeronautical Association, Türkkuşu Kampüsü, Ankara 06790, Turkey; 5 The Pritzker School of Molecular Engineering, The University of Chicago, Chicago, IL 60637, USA; mguler@uchicago.edu; 6 Institute of Materials Science, Vietnam Academy of Science and Technology, 18 Hoang Quoc Viet St., Hanoi 100000, Vietnam; hanhcong9389@gmail.com

**Keywords:** peptide amphiphiles, self-assembly, nanofiber scaffold, histidine, trace metals, reactive oxygen species, regenerative responses, neuronal differentiation

## Abstract

Synthetic peptides are promising structural and functional components of bioactive and tissue-engineering scaffolds. Here, we demonstrate the design of self-assembling nanofiber scaffolds based on peptide amphiphile (PA) molecules containing multi-functional histidine residues with trace metal (TM) coordination ability. The self-assembly of PAs and characteristics of PA nanofiber scaffolds along with their interaction with Zn, Cu, and Mn essential microelements were studied. The effects of TM-activated PA scaffolds on mammalian cell behavior, reactive oxygen species (ROS), and glutathione levels were shown. The study reveals the ability of these scaffolds to modulate adhesion, proliferation, and morphological differentiation of neuronal PC-12 cells, suggesting a particular role of Mn(II) in cell-matrix interaction and neuritogenesis. The results provide a proof-of-concept for the development of histidine-functionalized peptide nanofiber scaffolds activated with ROS- and cell-modulating TMs to induce regenerative responses.

## 1. Introduction

The development of biomaterial scaffolds with cell-supporting and regulating properties is of great importance in tissue engineering and regenerative medicine applications. Over the past few years, synthetic peptide-based materials have proved to be promising cellular matrices. The design versatility of peptide sequences along with their ability to adopt biomimetic spatial structure provide a flexible platform for the development of materials with controllable characteristics [1,2].

A number of strategies exist to develop supramolecular peptide biomaterials inspired by natural structures, including α-helices, β-sheets, and β-turns [3]. Other approaches propose the combination of peptide motifs with non-peptidic components such as polymers or hydrophobic segments to induce predicted self-assembly [4]. Particularly, peptide amphiphiles (PAs), in which hydrophobic groups are attached to hydrophilic peptide sequences, are capable of self-assembling into different nanostructures such as micelles, nanofibers, and nanotubes [4,5,6]. The self-assembly process is driven by non-covalent interactions including hydrogen bonding, hydrophobic and electrostatic interactions, and van der Waals forces [4,7].

The frequently used design of PAs produced by the solid-phase peptide synthesis technique consists of a short peptide sequence coupled with lauric (C_12_) or palmitic acid (C_16_) moiety [8,9]. For instance, C_16_-conjugated RGD-bearing PA nanofibers were shown to promote the adhesion, growth, and osteogenic differentiation of mesenchymal stem cells cultured on the material surface [10]. C_16_−KTTKS peptide stimulated the production of collagen in human dermal and corneal fibroblasts in a concentration-dependent manner [11]. Laminin epitope (IKVAV)-carrying C_12_-conjugated PA nanofibers efficiently induced neuronal differentiation and promoted neurite outgrowth in PC-12 cells [12]. The combination of C_16_−IKVAV and type I collagen in a hybrid matrix led to increased density and altered morphology of cultured neuronal Granule and Purkinje cells compared to the material formed by collagen alone [13]. To confer them with bioactivity, the peptide headgroups in most of the known PA constructs typically reproduce sequences derived from extracellular matrix (ECM) proteins involved in cell adhesion, survival, and signal transduction. A promising but barely investigated approach is the development of PAs with multi-functional moieties that, besides participating in the self-assembly process, permit the activation of the formed materials with bioactive agents such as metallic ions of interest to exploit their unique advantages for therapeutic applications. 

Trace metals (TMs) are essential microelements, which participate in vital enzymatic and non-enzymatic reactions involved in cell metabolism, functioning of the immune system, and turnover and regeneration of soft and hard tissues. The use of TMs in combination with biomaterial scaffolds is a promising tissue engineering strategy, where TMs may provide benefits over biological products in terms of stability, safety, and cost [14]. The regenerative potential of TM-based compounds and nanoparticles is supported by numerous studies [15,16,17,18].

The incorporation of Cu into mesoporous bioactive glass scaffolds promoted angiogenic and osteogenic differentiation of human bone marrow stromal cells by improving the expression of vascular endothelial growth factor and alkaline phosphatase, respectively [19]. Likewise, enhanced in vitro angiogenic responses of poly(2-hydroxyethyl methacrylate) materials modified with Cu (complexed with GHK peptide) were revealed according to the stimulation of proliferation, differentiation, and production of angiogenesis-related cytokines in human umbilical vein endothelial cells [20]. Zn-modified cryogels showed enhanced wound healing activity, accelerating the passing of the inflammatory/proliferation phase and dermis formation in a full-skin excisional model in rats [21]. Mn-containing bioactive glass ceramic material favored the differentiation of adipose tissue mesenchymal stem cells into adipogenic, chondrogenic, and osteogenic lineages [22].

Therefore, TMs are promising activating agents in self-assembled peptide matrices; however, the regenerative potentials of TM-modified PA scaffolds, particularly their roles in modulating cellular functions, have not been investigated. In this study, a series of PAs, C_n_-VVAGHH-NH_2_ (*n* = 12–17), containing β-sheet-forming sequence VVAG, hydrophilic di-histidine repeat, and a fatty acid tail were synthesized. Their self-assembly in nanofiber matrices promoted by deprotonation of histidine residues was elucidated using microscopic and rheological analyses. Furthermore, the feasibility of activation of PA gels with divalent TMs, Cu, Zn, and Mn via affinity complex formation with histidine residues was demonstrated. The modulating activity of the TM component toward mammalian cells was assessed in association with the change in ROS and reduced glutathione (GSH) levels, which are implicated in the redox regulation of multiple regeneration-related processes [23,24,25]. The effects of TMs on the morphological differentiation of PC-12 neuronal cells cultured on PA-based matrices were demonstrated.

## 2. Materials and Methods

### 2.1. Materials 

9-Fluorenylmethoxycarbonyl (Fmoc)-protected amino acids, fatty acids, Rink amide MBHA resin, and 2-(1H-benzotriazol-1-yl)-1,1,3,3-tetramethyluronium hexafluorophosphate (HBTU) were purchased from NovaBiochem. Other chemicals including dichloromethane (DCM), dimethylformamide (DMF), acetonitrile, piperidine, N,N-diisopropylethylamine (DIPEA), trifluoroacetic acid (TFA), and triisopropylsilane (TIPS) were purchased from Sigma-Aldrich (St. Louis, MO, USA).

4′,6-diamidino-2-phenylindole (DAPI), phenazine methosulfate (PMS), Triton X-100, and 2′,7′-dichlorofluorescin diacetate (DCFDA) were purchased from Sigma-Aldrich (St. Louis, MO, USA). Monochlorobimane (MCB) was purchased from ThermoFisher Scientific (Waltham, MA, USA). CuSO_4_·5H_2_O, ZnCl_2_, MnSO_4_·5H_2_O, and cresyl violet acetate were obtained from Acros Organics (Geel, Belgium).

3-(4,5-Dimethylthiazol-2-yl)-5-(3-carboxymethoxyphenyl)-2-(4-sulfophenyl)-2H-tetrazolium (MTS reagent) was purchased from Promega (Madison, WI, USA). Phalloidin CruzFluor™ 647 conjugate was purchased from Santa Cruz Biotechnology (Dallas, TX, USA). PC-12 and NIH 3T3 cell lines were obtained from the American Type Culture Collection (Manassas, VG, USA). Cell culture media and reagents were purchased from Paneco (Moscow, Russia).

### 2.2. PA Synthesis and Characterization

Peptide amphiphile molecules with varying lengths of hydrophobic alkyl tails were synthesized using the solid-phase fluorenylmethoxycarbonyl (Fmoc) peptide synthesis method. The synthesis of C_12_PA (C_12_-VVAGHH-NH_2_), C_13_PA (C_13_-VVAGHH-NH_2_), C_14_PA (C_14_-VVAGHH-NH_2_), C_15_PA (C_15_-VVAGHH-NH_2_), C_16_PA (C_16_-VVAGHH-NH_2_), and C_17_PA (C_17_-VVAGHH-NH_2_) peptides was carried out on Rink amide resin by iterative 2-h couplings of 2 mol equiv of a Fmoc-protected amino acid activated with 1.95 mol equiv of HBTU, and 3 mol equiv of DIPEA in DMF. The Fmoc protecting group was removed with 20% piperidine/DMF solution for 25–30 min. The peptides were cleaved from the resin with a mixture of TFA/TIPS/water in a ratio of 95:2.5:2.5 for 3 h and collected with DCM. DCM and excess TFA were removed by rotary evaporation. Then, peptides were precipitated using cold diethyl ether (−20 °C) and incubated overnight. The precipitate was collected by centrifugation. The centrifugate was dissolved in water, freezed at −80 °C and lyophilized for 3 days. Lyophilates were dissolved in 1 mM HCl solution and then freeze-dried again to remove any residual TFA. 

All peptides were purified using preparative high-performance chromatography. The identity and purity of the peptides were assessed by using Agilent 6530−1200 QTOF LC-MS with electrospray ionization source (ESI) equipped with a Zorbax SB-C18 column (rapid resolution HT 2.1 50 mm^2^, 1.8 μm). A gradient of A, 0.1% formic acid in water, and B, 0.1% formic acid in acetonitrile, was used in LC-MS analysis.

### 2.3. Preparation of the PA Nanofiber Gels

The PA powders were dissolved in Milli-Q water and sonicated for 15 min to obtain a homogeneous solution with a concentration of 1% (*w*/*v*). Then, PA gels were prepared by the addition of 10 µL NaOH (0.5 M) to 240 µL peptide solution in order to deprotonate the histidine residues (pK_a_ = 6.05).

Gels with metals were prepared in a 96-well plate. An aqueous solution of metal salts (CuSO_4_·7H_2_O, ZnCl_2_, or MnSO_4_·5H_2_O) was mixed with the PA solutions at a final metal concentration of 10 µM or 100 µM. To induce self-assembly, NaOH was added to the mixture solution (PA + metals) in the well.

### 2.4. Circular Dichroism (CD) Analysis

Previously prepared PA gels (11.5 mM PA solution) were diluted with water into 2 mM. Then, all samples were diluted to 0.3 mM. A Jasco J-815 CD spectrophotometer was used for CD analysis. Samples were measured between 190 and 300 nm with data pitch, 0.1 nm; sensitivity, standard; D.I.T., 4 s; bandwidth, 1 nm; scanning speed, 100 nm/min.

### 2.5. Transmission Electron Microscopy (TEM) Imaging

An FEI Tecnai G2 F30 TEM instrument was employed. Overnight incubated PA gels were diluted to 50 µM. A total of 10 μL of diluted sample was placed onto the TEM grid and the sample was kept on the grid for 8 min. Then, the excess sample was removed from the surface by a micropipette. Negative staining was performed using 2% (*w*/*v*) uranyl acetate for 2 min incubation. Then, the grid was immediately washed with double-distilled water and left to dry.

### 2.6. Scanning Electron Microscopy (SEM) Imaging 

One percent (*w*/*v*) peptide gels were prepared by using 0.5 M NaOH on a silicon wafer. After waiting 10 min for equilibrium, gels were incubated for 5 min in 20%, 40%, 60%, 80%, and 100% ethanol (overnight), respectively. Then, a critical point dryer was used to convert alcogel samples into aerogels. The morphologies of the samples were analyzed with an FEI Quanta 200 FEG scanning electron microscope. All samples were coated with 4 nm Au–Pd prior to imaging.

### 2.7. Atomic Force Microscopy (AFM) Imaging

Previously prepared PA gels were diluted into 150 µM solutions. Then, 10 µL from the solution was added onto glass and let dry. Dynamic and contact mode imaging were used to image the topography of the resulting samples using appropriate cantilevers. 

### 2.8. Oscillatory Rheology Analysis

Rheology measurements were performed with an Anton Paar Physica RM301 rheometer equipped with a 25 mm parallel plate at 25 °C. Previously prepared 11.5 mM peptide solutions (240 µL) were mixed with 0.5 M NaOH (10 µL) as a 250 µL total volume. The measuring distance was determined as 0.5 mm. Time sweep analysis of the samples was conducted at a constant 10 rad s^−1^ angular frequency and 0.01% strain to examine the viscoelastic characteristics of the nanofiber gels. The analysis of the gels was continued with an amplitude sweep test to reveal the linear viscoelastic range (LVR) at a constant angular frequency of 10 rad s^−1^ while logarithmically ramping the strain amplitude from 0.01% to 1000%.

### 2.9. Microcalorimetric Analysis

A microcalorimeter MicroCal PEAQ-ITC (Malvern, UK) was used to detect the formation of complexes between peptides and metals (each separately) within 50 min. Concentrations of 100 μM of PA solution and 4 mM metals (Mn, Cu, Zn) were used. The peptide solution and the metals were prepared in HEPES (50 mM) at pH = 5.5 to avoid gelation. To assess the binding of di-histidine (80 µM) with the metals (800 µM), the solutions of the peptide and metals were prepared in HEPES (50 mM) at pH = 7.4. 

### 2.10. Cell Maintenance and Seeding

PC-12 rat pheochromocytoma cells were maintained in DMEM supplemented with 10% HS and 5% FBS, penicillin (100 U/mL)/streptomycin (100 μg/mL), and L-glutamine (2 mM). NIH 3T3 mouse embryonic fibroblast cells were grown in α-MEM supplemented with 10% FBS, penicillin (100 U/mL)/streptomycin (100 μg/mL), and L-glutamine (2 mM). The cells were maintained in a humidity-controlled incubator under standard culture conditions (37 °C, 5% CO_2_). The culture medium was refreshed every 2 days.

Cells were seeded onto the surface of preformed PA matrices using the top seeding method in 96-well plate at a density of 5.1 × 10^4^ cells/cm^2^ of the material area and incubated for 1 h under standard culture conditions to allow for cell attachment. Cells were also cultured at the same density on the polystyrene surface of the tissue culture plate for comparison.

### 2.11. Cell Detection

To assess cell viability at 24 h post-seeding, the PA matrices with cultured cells were transferred into new wells containing 100 µL of MTS/PMS reagents in fresh culture medium and incubated for 1 h under standard culture conditions. The absorbance was then determined at 490 nm on an Infinite M200 PRO microplate analyzer (Tecan).

For bright-field microscopy analysis, the PA matrices with cultured PC-12 cells were fixed with 4% p-formaldehyde for 1 h, gently washed with PBS, and subsequently stained with cresyl violet (0.1% *w*/*v* in milli-Q water) for 5 min. The cells were visualized using an AxioObserver Z1 microscope (Carl Zeiss, Jena, Germany).

For cytoskeleton visualization, the fixed matrices were incubated in 0.1% Triton X-100 in PBS for 10 min for cell membrane permeabilization, followed by F-actin labeling using phalloidin CruzFluor™ 647 conjugate in 1% BSA for 20 min. The cell nuclei were stained with DAPI. LSCM images were acquired on an LSM 780 microscope.

### 2.12. Detection of ROS and Glutathione

The ability of TM-modified PA matrices to generate ROS extracellularly was studied by incubating the materials with H_2_O_2_ (100 µM) in PBS in the presence of DCFDA (5 µM). The kinetics of the reaction was assessed within a period of 30 min. Probe fluorescence (λ_ex_/λ_em_ = 490/526) in the solution was recorded on an Infinite M200 PRO microplate analyzer.

Intracellular effects of TM-modified PA matrices on both ROS and reduced glutathione (GSH) levels were additionally studied at 24 h post-seeding. The cell-seeded matrices were stained with DCFDA (20 µM, 40 min, λex/λem = 490/526) or with a monochlorobimane (MCB) probe (5 µM, 60 min, λex/λem = 380/480) to assess intracellular ROS and GSH, respectively.

The matrices with live cells co-stained with DCFDA/MCB were additionally visualized and analyzed using LSCM equipped with 488 nm argon and 405 nm diode lasers for dual excitation of DCFDA and MCB, respectively.

## 3. Results

### 3.1. Characterization of Self-Assembled Peptide Structures

**Figure 1:**
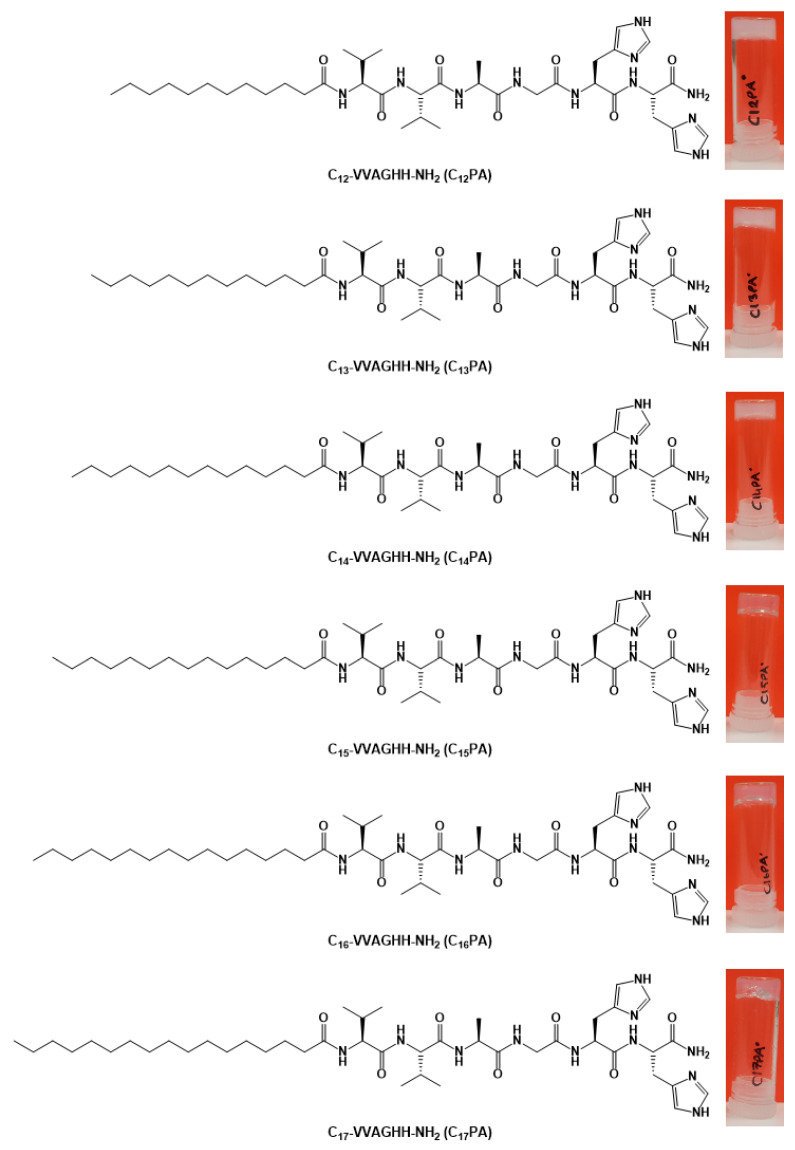
Structural formulas of the synthesized peptide amphiphiles and representative pictures of their corresponding gels.

The PA molecules consisting of the VVAGHH peptide sequence and fatty acid residue with varying aliphatic chains C_12_–C_17_ (Figure 1) were synthesized using Fmoc solid-phase peptide chemistry. The identity of synthesized PAs was verified by mass spectroscopy analysis (Figure S1). 

The aqueous self-assembly of PA into nanofiber structures mediated by the β-sheet-forming VVAG motif was triggered by increasing pH to cause deprotonation and a decrease in solubility of pH-sensitive HH segment [26]. Once formed, the PA gels are stable and non-dissolvable at physiological pH. The optical transparency of these gels was dependent on the alkyl tail length of PA. Relatively transparent and opaque white gels were obtained for PAs with longer (*n* ≥ 15) and shorter (*n* ≤ 14) alkyl tails, respectively. The light transmittance of the materials should be dependent on the colloidal properties of PA aggregates upon self-assembly.

**Figure 2:**
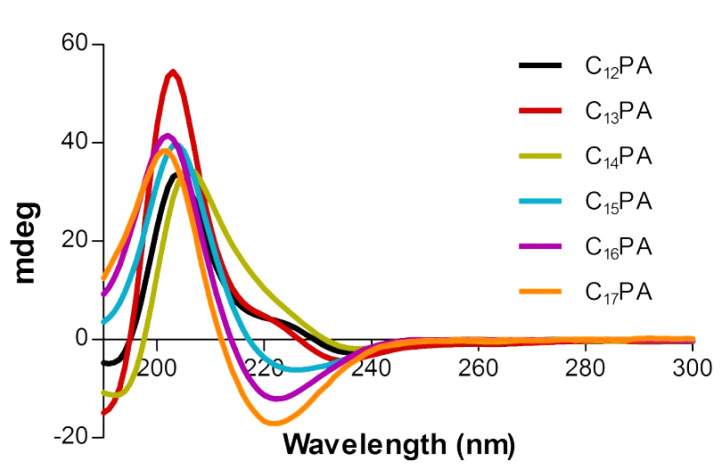
Circular dichroism (CD) spectra of PA assemblies (3 × 10^−4^ M).

To determine the secondary structure of the self-assembled PA nanostructures, circular dichroism (CD) spectroscopy was used. The typical β-sheet secondary structure has a characteristic bisignate CD spectrum due to the Cotton effect with a maximum and a minimum at ca. 195 and 215 nm, respectively [27]. All of the PA assemblies exhibited such a structure organization; the red-shifted bisignate signals observed between 195 and 240 nm indicated that the peptides assembled with twisted β-sheet structural motifs (Figure 2). The CD spectra of C_12_PA, C_13_PA, and C_14_PA assemblies showed a positive peak at ca. 205 nm, indicating the presence of a random coil structural motif. Random coil motifs may point toward the presence of oligomeric aggregates that affect gel transparency.

### 3.2. Microscopic Visualization

**Figure 3:**
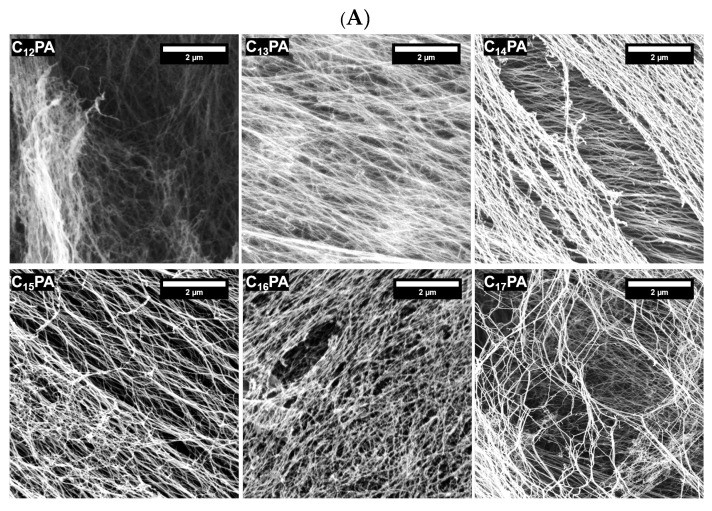

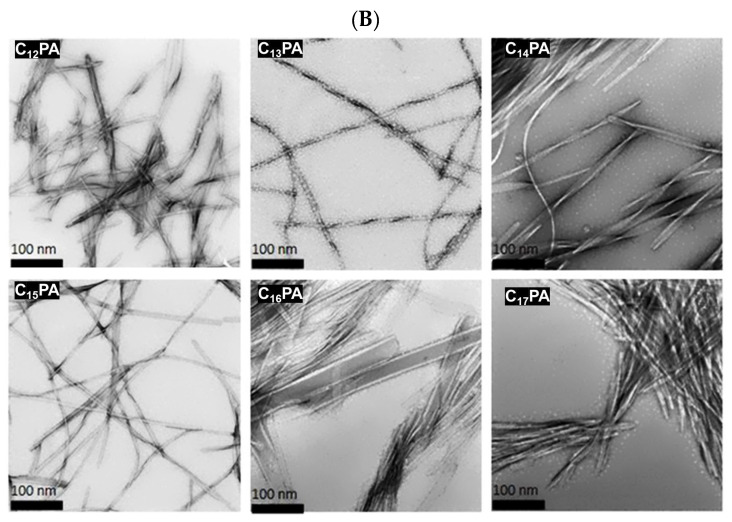
(**A**) SEM images of PA gel networks coated with 4 nm Au–Pd. (**B**) TEM images of PA nanofibers stained with 2% (*w*/*v*) uranyl acetate.

The three-dimensional PA gel networks were visualized and analyzed by AFM, SEM, and TEM techniques. According to SEM (Figure 3A) and AFM (Figure S2), all PA nanofibers formed bundles as a result of interfibrillar interactions. No significant differences in bundle and mesh size were observed between the compared nanofiber networks. TEM images demonstrated the formation of high-aspect-ratio nanofibers with diameters in the range 7–15 nm. (Figure 3B). While twisted ribbon-like structures were observed for PAs with a carbon tail length of *n* ≤ 15, no twisting was shown for C_16_PA and C_17_PA, demonstrating that longer alkyl tails of PAs more effectively dissipate torsional strain of twisted β-sheet stacks and enhance their ability to assemble. 

### 3.3. Rheological Analysis 

**Figure 4:**
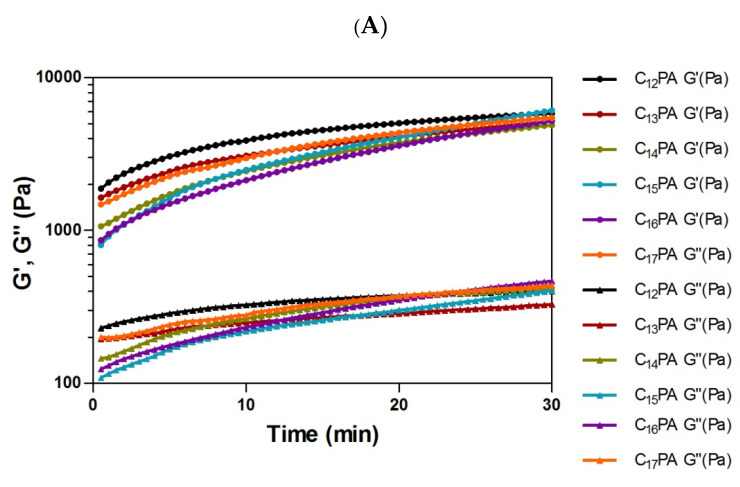

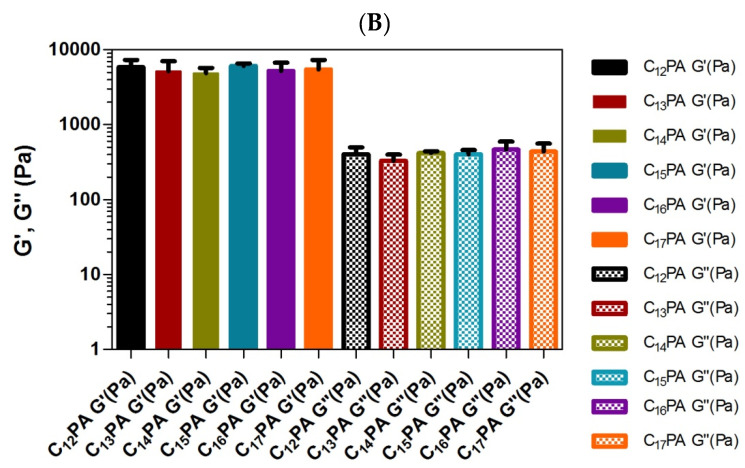
(**A**) Dynamic time sweep oscillatory rheology measuring the storage modulus (G′, filled circles) and loss modulus (G′′, filled triangles) of 1% (*w*/*v*) PA solutions after initiating self-assembly. (**B**) G′ and G′′ values of PA gels at equilibrium (mean ± SD, *n* = 3). The measurements were performed in viscoelastic region at constant strain (0.01%) and angular frequency (10 rad/s).

The viscoelastic properties of the PA gels were investigated using rheological analysis. The gelation kinetics of 1% (*w*/*v*) PA gels was monitored under constant angular frequency and strain. It was shown that all PA systems reached equilibrium within ca. 30 min (Figure 4A), and their storage modulus (G′) was more than 10 times higher than the loss modulus (G″), indicating a well-formed gel network with predominant elastic behavior (Figure 4B).

### 3.4. Thermodynamic Parameters of PA–Divalent Metals Complexation 

**Figure 5:**
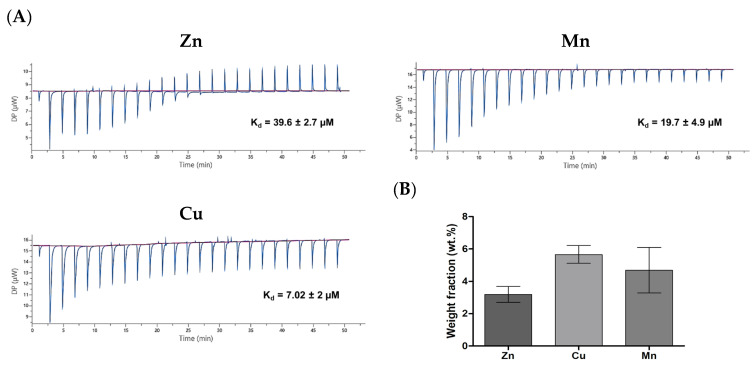
(**A**) Binding of di-histidine peptide to divalent metals (Cu, Zn, or Mn) according to isothermal titration calorimetry (50 mM HEPES, pH = 7.4, 37 °C). Integrated data for the titration after subtraction of control are shown. (**B**) SEM-EDX analysis of elemental composition of TM-modified C_12_PA gels.

The complexation of the peptides and metal ions was studied using the ITC technique based on the detection of heat discharge or consumption upon intermolecular interactions [28]. The analysis showed that the PAs exhibited affinity to the divalent TMs attributed to the presence of metal-binding histidine residues; for instance, the detected dissociation constants (K_d_) for C_12_PA were as follows: 6.8 μM (Zn), 3.6 μM (Mn), and 2.8 μM (Cu) (Figure S3). However, fatty acid residues may significantly interfere with the heat effects upon the interaction of amphiphilic peptides due to concurrent hydrophobic association [29]. Therefore, the interaction of HH dipeptide with the TMs was additionally assessed, providing corrected K_d_ values for the metal-binding segment of PAs: 39.6 µM (Zn), 19.7 µM (Mn), and 7 µM (Cu) (Figure 5A). This confirms the ability of the histidine repeat to form a coordination complex with the divalent TMs at sufficiently high affinity to insure their attachment to the PA matrices. 

Energy dispersive X-ray spectroscopy (EDS) analysis was conducted to analyze the elemental composition of the TM-containing PA gel matrices, which were prepared by mixing PA and TM solutions prior to gelation. The EDS analysis showed that the TMs were successfully incorporated into the PA materials (Figure 5B and Figure S4). The detected weight fractions of the analyzed metals were in the following order: 3.2 wt.% (Zn), 4.7 wt.% (Mn), and 5.6 wt.% (Cu), which is in general agreement with the corresponding affinity as indicated by K_d_ values.

### 3.5. Cell-Modulating Effects of TM-Modified PA Matrices

#### 3.5.1. Cell Adhesion and Proliferation

PC-12 rat pheochromocytoma cells were used as a neuronal cell model to assess the biocompatibility and biological activities of the materials [30,31]. The cells were seeded onto the surface of formed PA gels to assess cellular behaviors, which relate to cell adhesion, proliferation, and differentiation [32,33]. It is noteworthy that irrespective of the tail length, all the PAs formed fairly similar three-dimensional matrices with slight difference in the fibrillar structure and rheological characteristics (Figure 4). In consistency with that, mammalian cells had similar behavior on the studied PA materials in the absence of complexed TMs. Therefore, cellular effects of TM-modified PA matrices were demonstrated on the example of C_12_PA-based gels.

**Figure 6:**
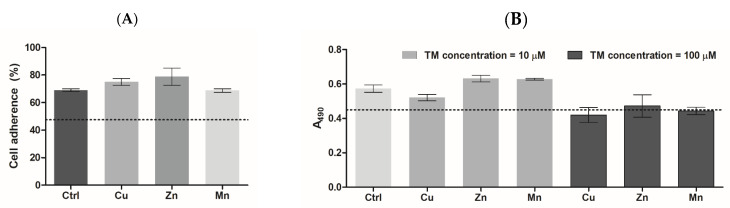
(**A**) Adherence of PC-12 cells on the surface of trace metal-modified PA matrices after 4 h incubation (% of the total cell number). (**B**) Viability of PC-12 cells cultured on trace metal-modified PA matrices (metal concentration was 10 or 100 µM), according to MTS test, 24 h post-seeding. The dotted line shows the cell adherence (%) (**A**) or cell viability signal (**B**) on the polystyrene surface of tissue culture plate.

The PA gels supported a significantly higher primary adhesion of PC-12 cells compared to the cell culture polystyrene surface (*p* < 0.5) with detected adhesion rates of ca. 48 and 70%, respectively (Figure 6A). This is explained by increased cell interaction with the surface covered with PA nanofibers. The presence of TMs did not alter the adherence of PC-12 cells, which was in the range of 70–79%.

According to the MTS assay (24 h post-seeding), PC-12 cells cultured on PA gels exhibited an increased proliferation of 30 ± 6% compared to those cultured on the polystyrene surface (Figure 6B). At a concentration of 10 µM, the introduced TMs tended to modulate cell viability/proliferation with weak inhibitory or stimulating effects, respectively, for Cu and Zn/Mn. At a concentration of 100 µM, all the metals tended to slightly inhibit the cells compared to metal-free PA (Ctrl), but without displaying any cytotoxic effect compared to a polystyrene surface.

Compared to PC-12 cells, 3T3 fibroblasts cultured on PA materials showed somewhat different behavior (Figure S5). Their proliferation was found to be less supported on the PA matrices vs. polystyrene surface. This could be associated with distinct type-specific cell adhesion behaviors on the PA surfaces, where the positive charge provided by the imidazole groups is, apparently, more relevant for neuronal cells.

Taken together, these data show the lack of cytotoxicity of the PA-incorporated TMs at a micromolar range of concentration (particularly, at C ≤ 10 µM) and confirm the cytocompatibility of the materials and their ability to support cell viability and growth. The cell-modulating effects of the metals may be associated with their redox activity.

#### 3.5.2. ROS-Modulating Effects of TM-Modified PA Matrices

**Figure 7:**
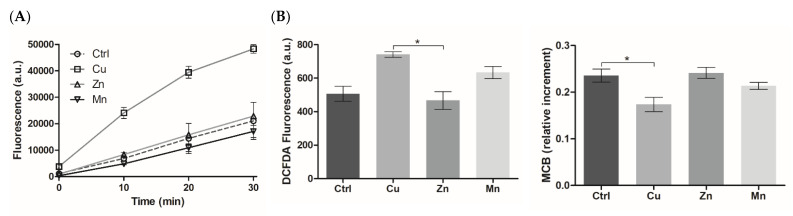

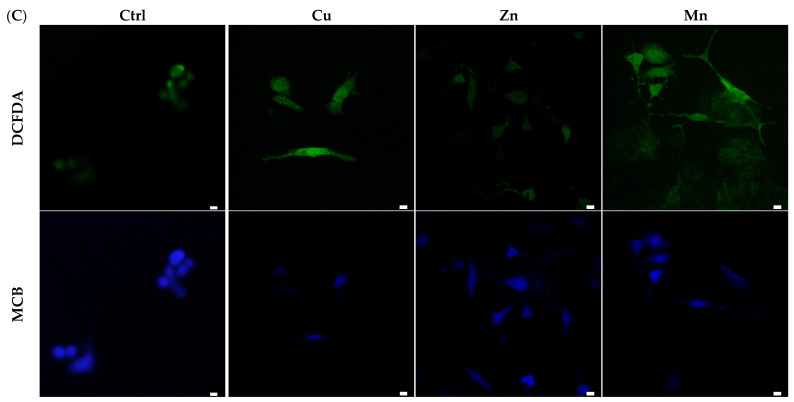
(**A**) Extracellular generation of ROS in the reaction of TM-modified PA matrices with H_2_O_2_ according to DCFDA fluorescence (λex/λem = 490/526 nm). (**B**) Intracellular levels of ROS and GSH in PC-12 grown on TM-modified PA matrices (metal concentration was 10 µM) at 24 h post-seeding (* *p* < 0.05). The levels of GSH were determined according to monochlorobimane (MCB) fluorescence (λex/λem = 380/480). (**C**) LSCM of TM-modified PA matrices with live cells co-stained with DCFDA/MCB. Argon (488 nm) and diode (405 nm) lasers were used for dual excitation of DCFDA and MCB, respectively. Scale bar 20 µm.

The ability of the TM-modified PA materials to generate ROS in the reaction with hydrogen peroxide (H_2_O_2_) was analyzed using a DCFDA probe (Figure 7A) considering the role of ROS as a secondary messenger in regeneration-related cellular responses [34]. The results showed that Cu, unlike the other TMs, considerably promoted ROS formation (*p* < 0.5) compared to TM-free material in accordance with earlier observation for Cu-modified versus Zn-modified gelatin cryogels [14], reflecting the H_2_O_2_–mediated pro-oxidant activity of copper ions (II). Similarly, manganese ions (II) in a soluble form were also shown to participate in the Fenton-like reaction [35]; however, the quite different behavior of Cu and Mn components in the PA matrices can be attributed to the different effects of histidine ligands on the reactivity of TMs.

Furthermore, intracellular levels of ROS and reduced glutathione (GSH), which reflect the oxidative state of the cells [36], were detected in living PC-12 cells grown on the PA gels (Figure 7B). According to the microplate analysis, these levels were differently influenced by the TMs. The Cu component (10 μM) induced ROS overproduction accompanied by some decrease in GSH content, suggesting an increased ability of Cu to elevate the oxidative state of the cells (without causing any cytotoxicity). Such an ability was less significant in the case of the Mn component, whereas the Zn component showed a lack of pro-oxidant activity. The effects of PA-formulated TMs were additionally confirmed using LSCM analysis of MCB- and DCFDA-stained cells (Figure 7C). 

#### 3.5.3. Cell Morphology

**Figure 8:**
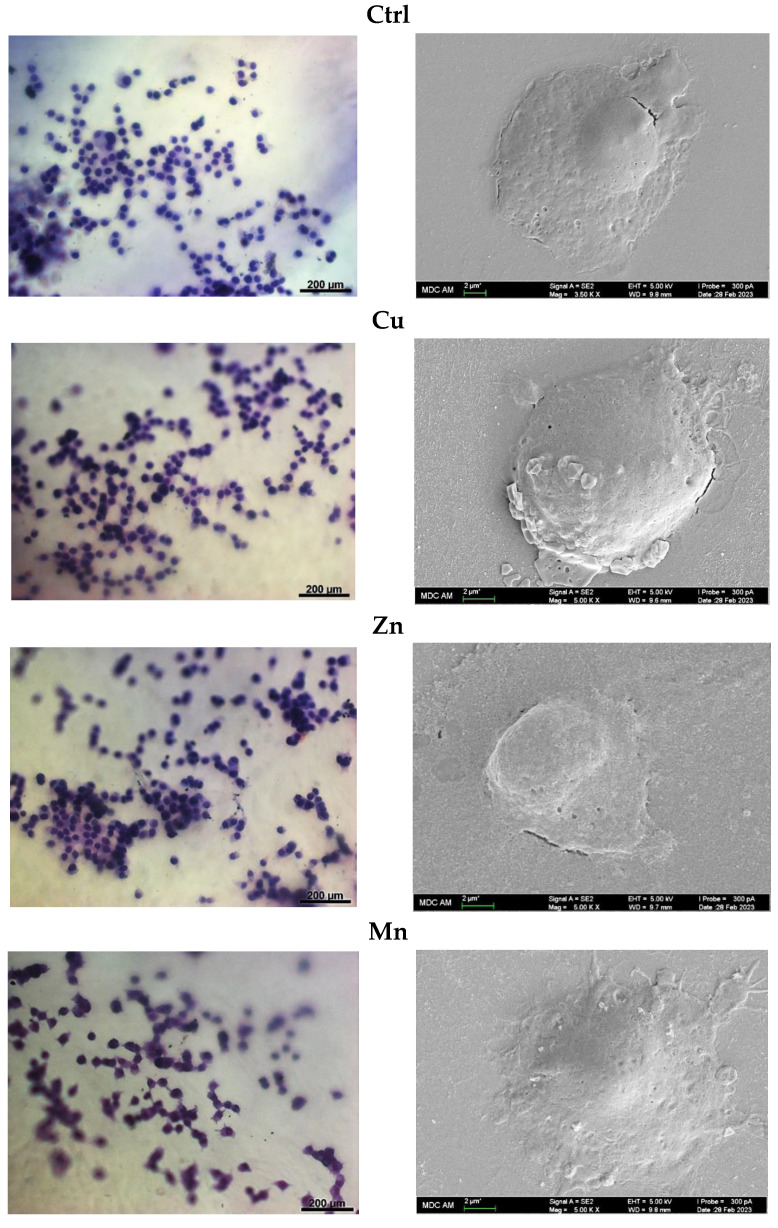
Bright-field microscopy images (**left panel**) and scanning electron microscopy images (**right panel**) of fixed PC-12 cells at 24 h post-seeding on TM-modified PA matrices. For bright-field microscopy, the cells were stained with cresyl violet.

The morphology of PC-12 cells grown on the PA gels with TMs was assessed using bright-field microscopy (BFM), SEM, and LSCM. The LSCM analysis with phalloidin CruzFluor™ 647 conjugate was strongly interfered with by the probe adsorption on the peptide film (Figure S6) whereas BFM with cresyl violet allowed for cell visualization on the matrices. It was found that the blank PA material effectively supported cell attachment and growth. No distinct difference existed in cell appearance between metal-free PA- (Ctrl), Zn-PA, and Cu-PA materials, where the cells mostly showed round morphology typical of non-differentiated cells (Figure 8). However, the cells grown on the surface of the Mn-PA gel were characterized by better spreading and emerged extensions attributed to neuritogenesis upon differentiation to neurone-like cells [37]. More than half of the cultured cells (57 ± 5%) displayed neurite extensions, while for 18 ± 2% of them, a mean neurite number ≥ 3 was detected. A number of the adjacent cells showed a tendency to connect to each other with neurite lengths up to 21 ± 5 µm (Figure 8). In addition, the SEM analysis confirmed enhanced spreading and neuritogenesis of PC-12 cells on the Mn-PA gel (Figure 8).

## 4. Discussion

C_n_–VVAGHH–Am amphiphilic peptide sequences were designed as biomimetic nanofiber-forming PAs with pH-dependent self-assembling ability and affinity toward bioactive TMs. The self-assembly behavior of PAs was influenced by the alkyl tail (*n* = 12–17), which modulated the secondary structure of the peptide molecules (Figure 2) and the number of twisted nanofibers (Figure 3) but provided similar gelation ability irrespective of the tail length. As shown previously, the hexyl group when coupled to CCCCGGGS(PO_4_)RGD peptide failed to form a stable gel under slow acidification conditions unlike longer chained counterparts (*n* ≥ 10) [38]. Increasing the pH value from 7 to 11 of a solution of RGD-based PAs resulted in the collapse of nanofiber structures formed by C_9_-conjugates, whereas the structures formed by C_13_- and C_15_-conjugates were stabilized by increased hydrophobic interactions of the longer alkyl tails [39]. According to our results, all hydrophobic tails used in the sequences (*n* = 12–17) ensured the formation of stable nanofiber gels with similar viscoelastic behavior (Figure 4), suggesting no critical transition of amphiphilic and self-assembling properties of PAs in the range of hydrophobic segment length.

The di-histidine repeat introduced into the PA sequence plays dual structural and functional primary roles. At acidic pH, the PAs have a positive net charge due to the presence of imidazole groups (pK_a_ of histidine imidazole is 6.05). Deprotonation of these groups upon alkalinization and concomitant reduction in the electrostatic repulsion of PA molecules promotes their assembly into nanofibrillar structures [40]. In particular, PA charge neutralization favors the formation of β-sheets and leads to ultimate changes in the spatial structure of PA ensembles, e.g., as a result of the merging of spherical micelles. The latter could be formed at lower pH conditions as an intermediate system essentially driven by hydrophobic interactions between alkyl tails [41].

The other function of the di-histidine segment is the complex formation with the TMs. As revealed by ITC and elemental analysis data (Figure 5 and Figure S3), the PA molecules and gels effectively bound the divalent Cu, Zn, and Mn ions with relatively high affinity. An important role of histidine-containing peptide ligands in the imidazole-mediated formation of coordination bonds with TMs is well-established for different natural proteins and peptides [42,43,44]. For instance, in superoxide dismutase (SOD1), divalent Cu and Zn metals are kept in place and in close proximity by six histidine residues [45]. Besides nitrogens in the imidazole group of histidine, amide bonds can also participate in the chelation process at physiological pH of 7.4, through deprotonation of the bond and the formation of a negatively charged amidyl ligand. Well-known examples are Cu(II)-binding GHK (Xaa-His) and DAHK (Xaa-Yaa-His) peptides where one and two amidyl ligands contribute to the binding, respectively [46]. Previously, the ability of the GHK peptide (Ada-Ahx-GGGHK-NH_2_) to form a complex with Cu(II) was used to activate macroporous cryogels to stimulate the proliferation and differentiation of human umbilical vein endothelial cells [20].

The cell-supporting ability of the formed TM-formulating PA gels was investigated. The enhanced PC-12 cell adherence shown on the surface of the PA gels can be explained by some cationic charge of histidine residues, which is known to play an important role in the primary attachment of neuronal cells to the matrix [47]. The lack of cytotoxicity of the formulated TMs may indicate minimal release of these components into the cell culture medium during the studied period. In consistency with this observation, cryogel-incorporated metals were partially available to the cells upon culturing and did not display cytotoxicity compared to their soluble counterparts at a relatively high concentration of up to 200 µM [14,21]. Likewise, decreased cytotoxicity was reported for TMs in bioactive glasses [19,48]. 

The analysis of cell morphology demonstrated that cell attachment alone is not a sufficient parameter for the induction of cell differentiation, which was found to be specifically dependent on the used TM, highlighting an adjunct role of divalent Mn for such a process. In agreement with these results, the treatment of PC-12 cells with Mn^2+^ (MnCl_2_) promoted cell spreading and the extension of neurite-like structures in a time- and dose-dependent manner, whereas other tested metals (Mg^2+^, Cd^2+^, Cu^2+^, Zn^2+^, and Ni^2+^) failed to induce neurite outgrowth [49]. However, unlike the cells cultured on Mn-modified PA materials (Figure 8 and Figure S6), the cells treated with MnCl_2_ lacked the ability to form networking of the emerged neurites [49].

The morphological changes induced by Mn resemble those produced during the initial phase of NGF treatment; however, the cell response to Mn occurs faster (several hours vs. several days) [37]. A potential mechanism of Mn activity is the upregulation of surface expression of α_v_ integrins such as vitronectin receptor (α_v_β_1_) [50]. Moreover, it was reported that Mn-induced neurite outgrowth is initiated by its interaction with integrins and vitronectin or fibronectin basement membrane proteins [37,50]. Mn^2+^ are known to bind integrin receptors and increase their affinity toward RGD ligand, known as the minimal essential sequence of cell binding in the aforementioned proteins [5]. At a concentration as low as 10 μM, Mn^2+^ was able to stimulate neurite formation in PC-12 cells cultured on substrata coated with either fibronectin or vitronectin [37]. The induction of neuritogenesis by Mn is accompanied by increased cellular levels of the neuronal-specific marker proteins, peripherin and GAP-43 [37,49]. The effects of Mn could be also related to its competition with Ca ions for calmodulin binding, leading to neuronal differentiation by changing the concentration of intracellular calcium [51]. However, high concentrations of Mn can provoke ATP depletion for the compulsory transport of a large amount of Mn^2+^ through calcium channels, which further contributes to cell death via caspase-dependent apoptosis or caspase-independent necrosis [24].

The data support specific ROS-modulating activities of the PA-formulated TMs (Figure 7), demonstrating that the pro-oxidant effects of Cu generally prevailed over those of Mn and Zn. Given that both free Mn and Cu ions can participate in Fenton-type reactions [35], the lack of ROS generation by H_2_O_2_ by the Mn-PA system compared to the Cu-PA system (Figure 7A) suggests a different role of the peptide ligand in the reactivity of the TMs. It was previously revealed that Zn ions, despite their inhibitory effect on the Fenton-type reaction in the extracellular system, were able to induce a moderate pro-oxidant effect intracellularly [14]. This indicates that the redox-modulating properties of TMs apparently depend on the conditions and metabolism of the target cells. Compared to formulated TMs, more pronounced pro-oxidant activities of soluble counterparts with distinct mechanisms of action were shown earlier [14]. While the effects of Zn were attributed to their interaction with multiple thiol groups of Zn-binding cysteine-rich proteins [25] and activation of mitochondrial lipoamide dehydrogenase [52], the effects of soluble Cu were due to Fenton-like reaction [35] and redox cycling with biomolecules [53,54]. Lowering of the cytotoxic and pro-oxidant effects of these ions complexed in PA gels probably results from the reduction in their cellular transportation/availability, which minimizes side reactions.

The effects of the Mn component on neuronal cell differentiation of PC-12 cells on the PA matrix (Figure 8 and Figure S6) could be mediated by induction of low ROS levels (Figure 7), where the metal–peptide complexation could provide a continuous release and cellular delivery of Mn ions. The use of gold nanoparticles for cellular delivery of Mn ions in a sustained manner allowed the avoidance of mitochondrial damage and cell necrosis caused by unformulated Mn^2+^ and resulted in improved neuronal differentiation of PC-12 cells [51]. Similarly, the inclusion of Mn in the PA matrices helped to achieve neuromodulating activity while avoiding the neurotoxic effects of high Mn concentration, which may disrupt cellular metabolisms. 

A fine balance exists between ROS levels and the induced cellular responses. Abnormal production of ROS can lead to the depletion of GSH, underlying a series of pathological conditions including chronic inflammation and degenerative diseases such as Alzheimer’s or Parkinson’s disease [55,56], whereas at subtoxic levels, ROS act as modulators of gene transcription and activators of signaling pathways contributing to cellular proliferation and differentiation [57,58]. Moreover, a neuroprotective role of subtoxic levels of ROS was reported, showing their ability to induce both the expression of brain-derived neurotrophic factor (BDNF), significantly attenuating PC-12 cell death [34,59], and BDNF-independent activation of TrkB (BDNF receptor) in neuronal cells [60].

## 5. Conclusions

We demonstrated a rational design of histidine-containing peptide amphiphiles forming self-assembling nanofiber scaffolds for metal coordination and cell behavior modulation. The histidine repeat was exploited both to provide pH-dependent peptide association and activation of the scaffolds with divalent Zn, Cu, and Mn trace metal ions via coordination complex formation. Stable PA-based hydrogel matrices with similar viscoelastic behavior and three-dimensional fibrillar structure were produced, generally irrespective of the hydrophobic tail length of the peptides (*n* = 12–17). The cytocompatibility of the metal-modified PA matrices at micromolar TM concentrations was confirmed, and their modulating effects on mammalian cell behavior were assessed in relation to pro-oxidant effects. The TM-activated PA scaffolds were proven to be supportive of cell adhesion, viability, and growth. A particular ability of Mn to induce morphological differentiation of PC-12 neuronal cells was demonstrated. Altogether, the reported data provide a proof-of-concept for the development of TM-activated PA-based matrices as a promising biomaterial for tissue engineering and regeneration applications.

## Data Availability

The data presented in this study are contained within the article and Supplementary Materials.

## Supplementary Materials

The following supporting information can be downloaded at: https://www.mdpi.com/article/10.3390/mi14040883/s1, Figure S1: Mass spectra of synthesized peptide amphiphile molecules. Figure S2: Atomic force microscopy images of self-assembled peptide amphiphile structures: (a) C_12_PA, (b) C_13_PA, (c) C_14_PA, (d) C_15_PA, (e) C_16_PA, and (f) C_17_PA. Figure S3: Binding of amphiphilic peptides to divalent metals (Cu, Zn, or Mn) according to isothermal titration calorimetry (50 mM HEPES, pH = 5.5, 37 °C). Integrated data for the titration after subtraction of control are shown. Figure S4: SEM-EDX analysis of the elemental composition of TM-modified PA gels. Figure S5: A. Adherence of 3T3 fibroblasts on the surface of trace metal-modified PA matrices after 4 h incubation (% of the total cell number). B. Viability of 3T3 fibroblasts cultured on trace metal-modified PA matrices (metal concentration was 10 or 100 µM), according to MTS test, 24 h post-seeding. The dotted line shows the cell adherence (%) (A) or cell viability signal (B) on the polystyrene surface of the tissue culture plate. Figure S6: Images of fixed PC-12 cells on TM-modified PA matrices visualized at 24 h post-seeding using bright-field microscopy (A), scan electron microscopy (B), and laser scan confocal microscopy (C). The cells were stained with cresyl violet (bright-field microscopy) or phalloidin CruzFluor™ 647 conjugate (LSCM) prior to visualization.
