# Supplementary material for: Metal-Chelating Self-Assembling Peptide Nanofiber Scaffolds for Modulation of Neuronal Cell Behavior
Source: Micromachines (Basel). 2023 Apr 19;14(4):883. doi: 10.3390/mi14040883 (PMC10146964; doi:10.3390/mi14040883)
Supplement: Supplementary file 1 [file micromachines-14-00883-s001.zip › micromachines-2360232-supplementary.pdf]

micromachines

Supplementary Material

Metal Chelating Self-assembling Peptide Nanofiber Scaffolds for Modulation of Neuronal Cell Behavior

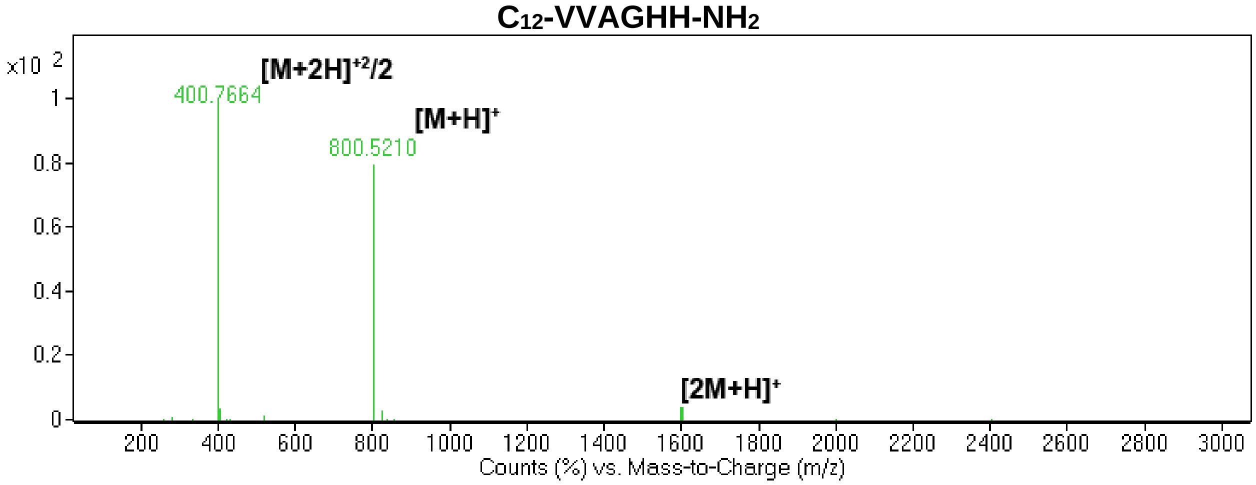

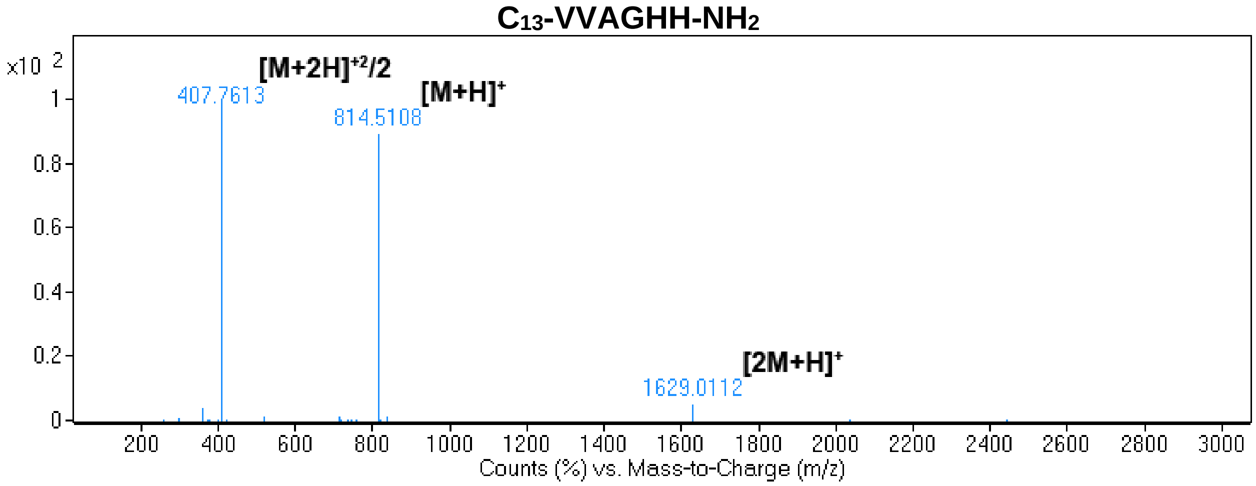

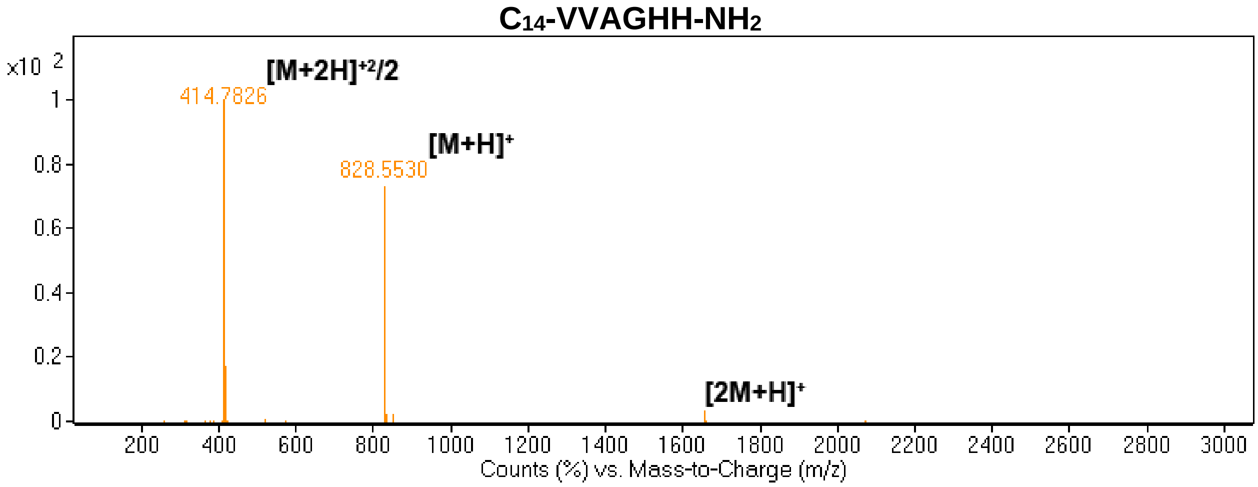

C_{15}-VVAGHH-NH_2

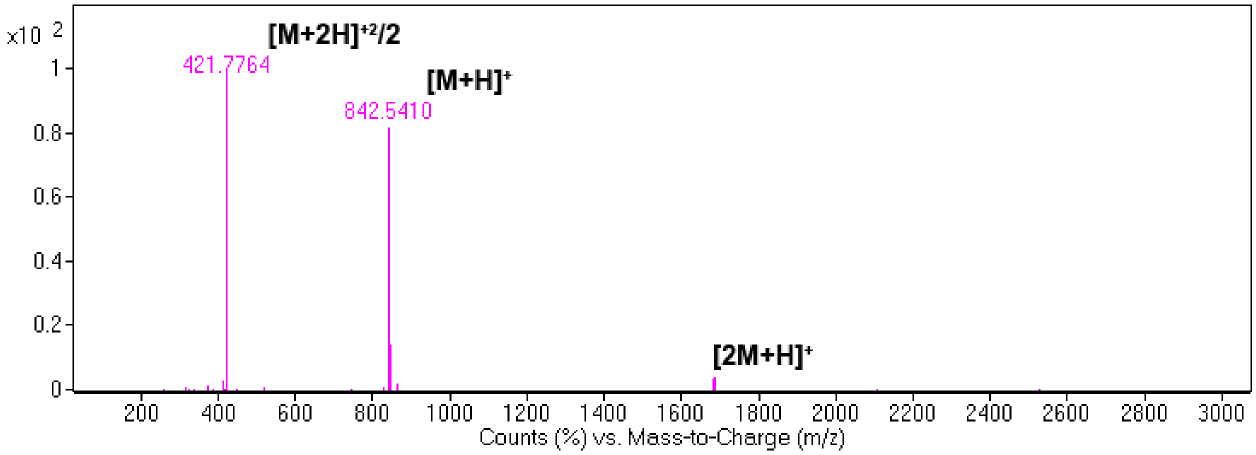

C_{16}-VVAGHH-NH_2

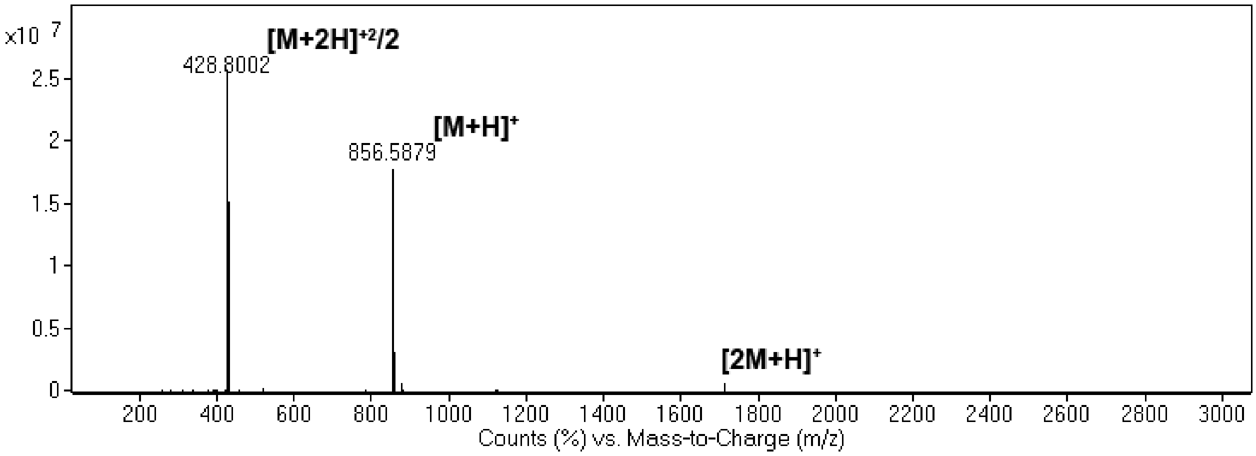

C_{17}-VVAGHH-NH_2

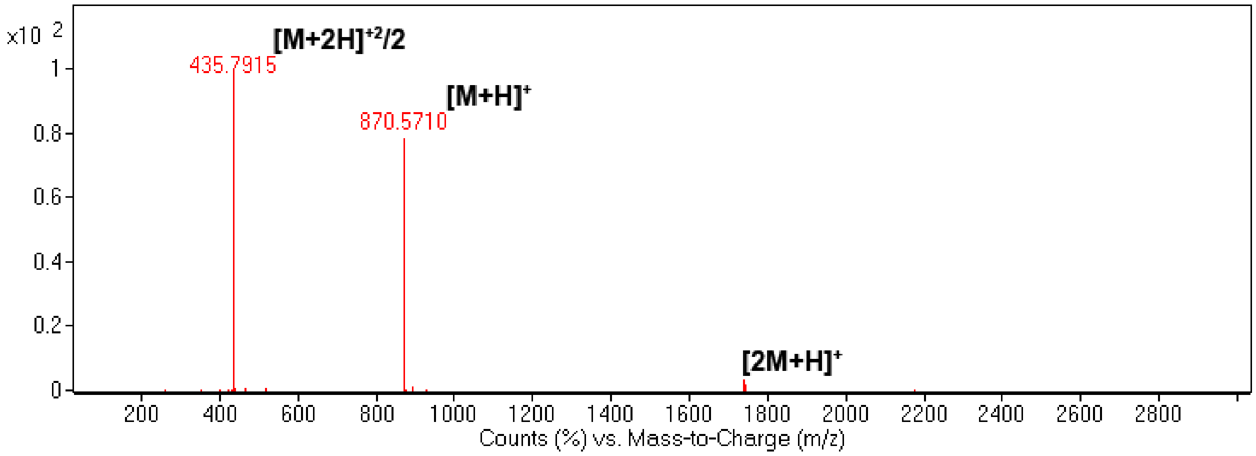

Figure S1. Mass spectra of synthesized peptide amphiphiles. The m/z values corresponding to $[M+H]^+$ (target peak), $[M+2H]^{+2}/2$, and $[2M+H]^+$ ionized species are detected.

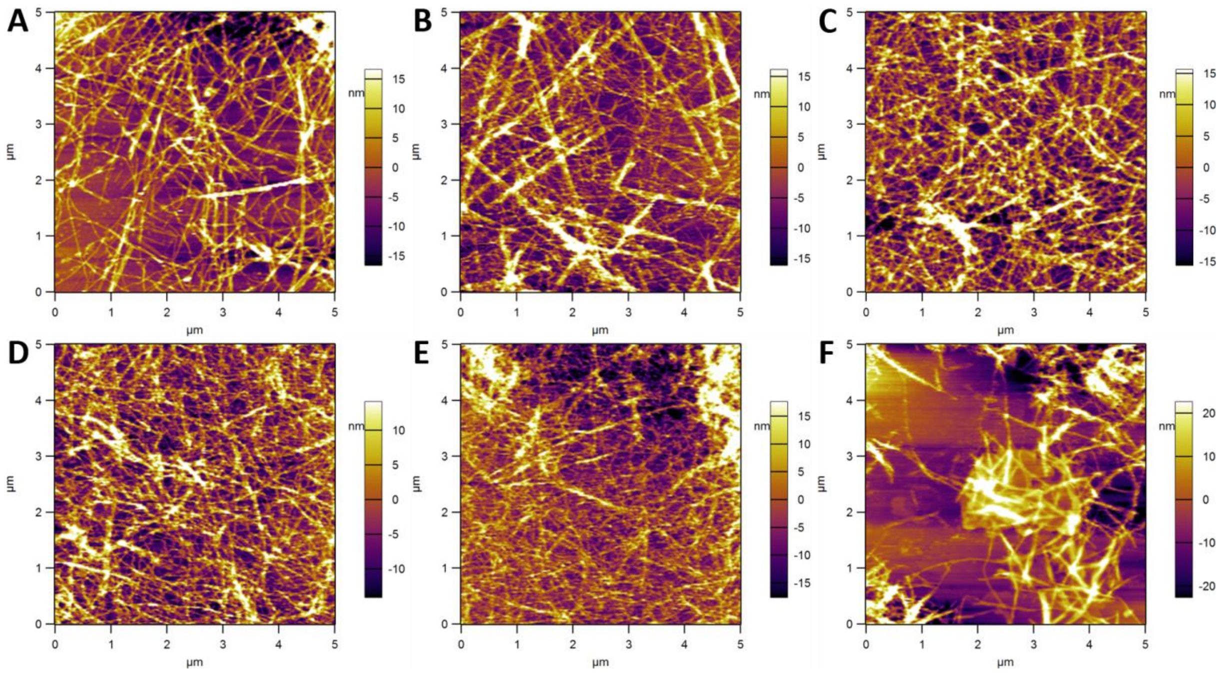

Figure S2. Atomic force microscopy images of C_{12}PA (A), C_{13}PA (B), C_{14}PA (C), C_{15}PA (D), C_{16}PA (E), and C_{17}PA (F).

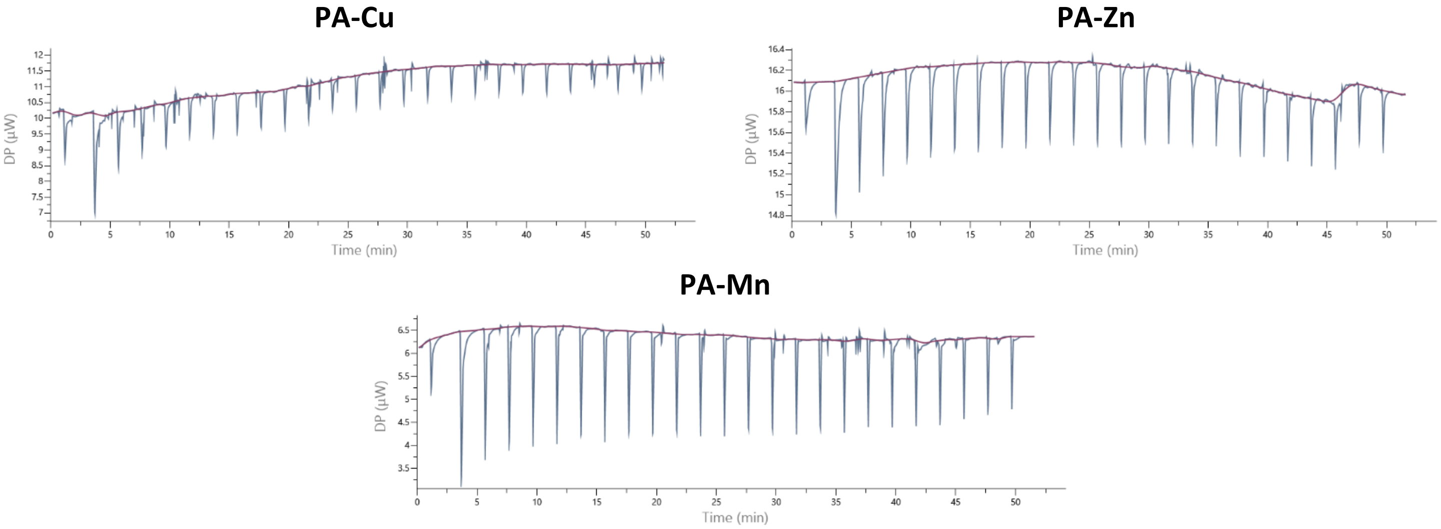

Figure S3. Binding of amphiphilic peptides to divalent metals (Cu, Zn, or Mn) according to isothermal titration calorimetry (50 mM HEPES, pH = 5.5, 37 °C). Integrated data for the titration after subtraction of control are shown.

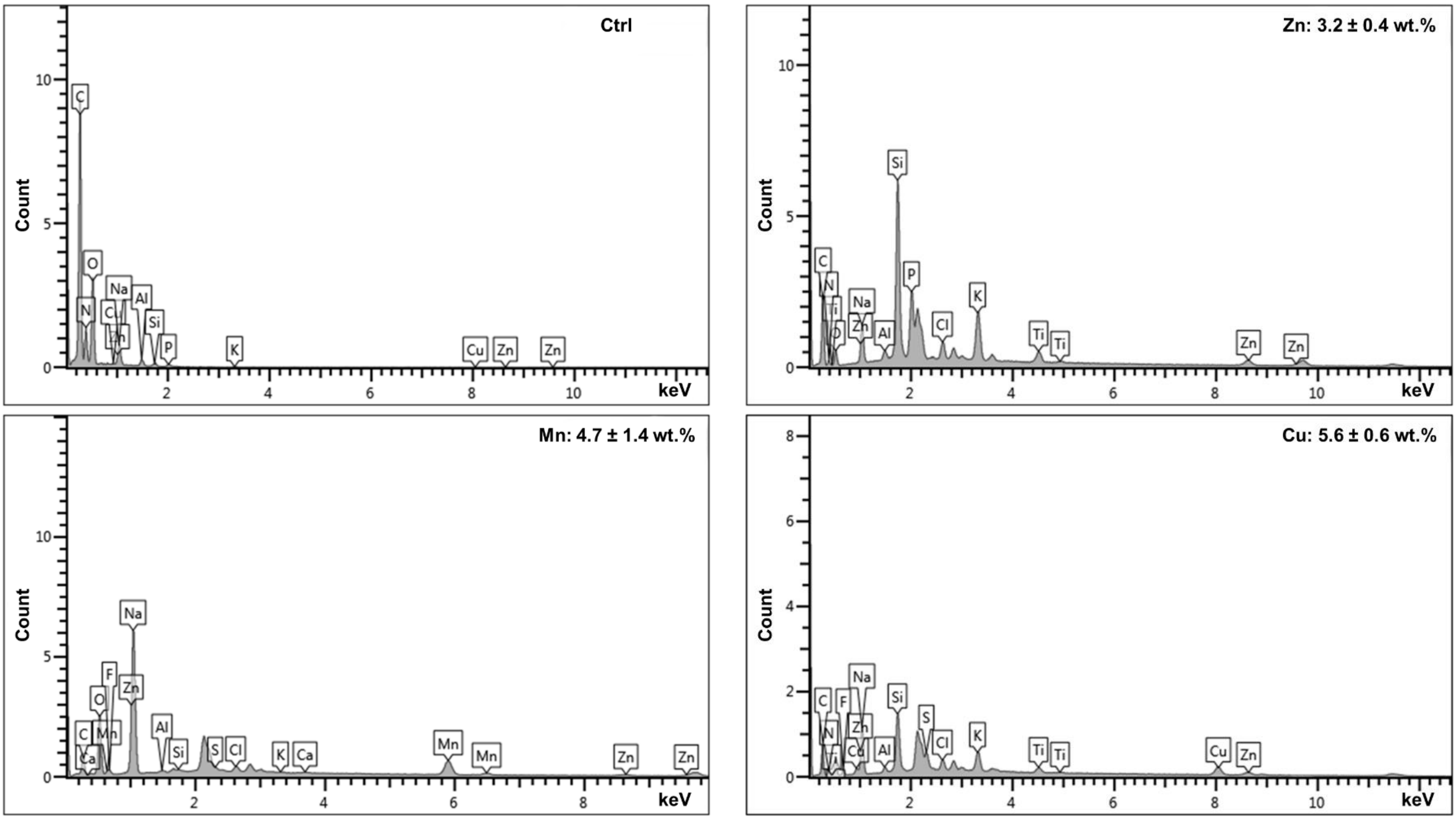

Figure S4. SEM-EDX analysis of elemental composition of TM-modified PA gels.

A

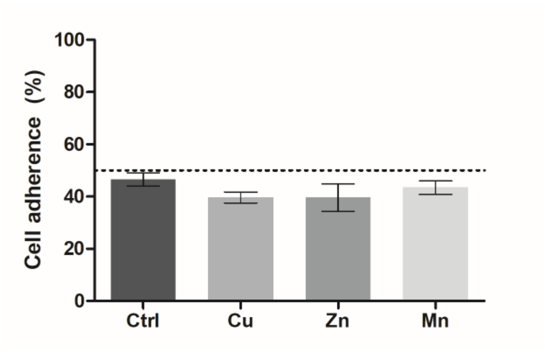

B

micromachines

MDPI

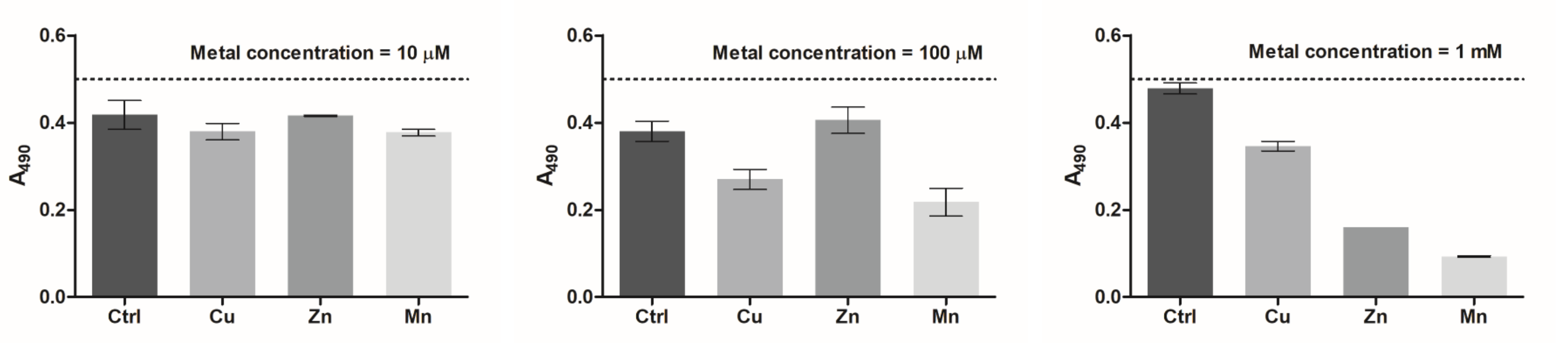

Figure S5. A. Adherence of 3T3 fibroblasts on the surface of trace metal-modified PA matrices after 4 h incubation (% of the total cell number). **B.** Viability of 3T3 fibroblasts cultured on trace metal-modified PA matrices (metal concentration was 10, 100, or 1000 µM), according to MTS test, 24 h post-seeding. Dotted line shows the cell adherence (%) (A) or cell viability signal (B) on the polystyrene surface of tissue culture plate.

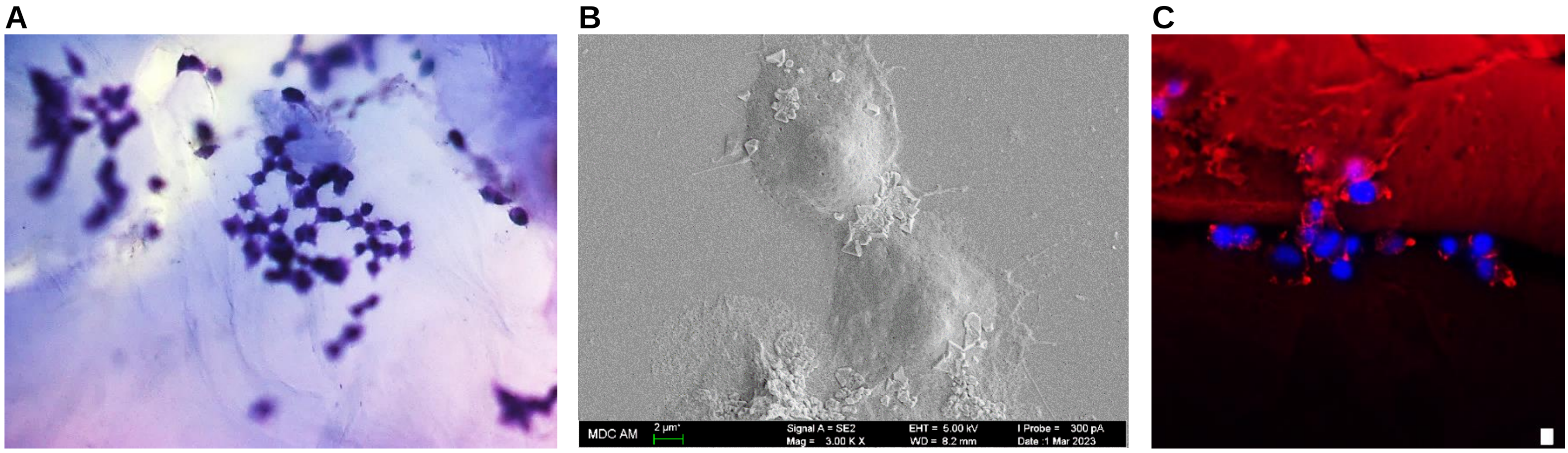

Figure S6. Images of fixed PC-12 cells on TM-modified PA matrices visualized at 24 h post-seeding using bright-field microscopy (**A**), scan electron microscopy (**B**), and laser scan confocal microscopy (**C**). The cells were stained with cresyl violet (bright-field microscopy) or phalloidin CruzFluor™ 647 conjugate (LSCM) prior visualization.
